# The intention for cesarean section scale: Turkish validity and reliability study

**DOI:** 10.1590/1806-9282.20251499

**Published:** 2026-05-01

**Authors:** Seçil Hür, Sefa Karaman, Elif Dağlı, Ayça Şolt Kırca

**Affiliations:** 1 Kırklareli University, Faculty of Health Sciences, Department of Midwifery – Kırklareli, Türkiye.; 2 Çukurova University, Abdi Sütcü Vocational School of Health Services, Department of Health Care Services – Adana, Türkiye.

**Keywords:** Cesarean section, Intention, Reliability, Validity

## Abstract

**OBJECTIVE:**

In recent years, cesarean section rates have been increasing worldwide. Identifying changes in birth preferences during pregnancy is crucial for reducing cesarean section rates.

**AIM:**

The aim of this study was to determine the Turkish validity and reliability of the Theory-Based Intention for cesarean section scale, originally developed by Naghibi et al.

**METHODS:**

This methodological study was conducted with 300 pregnant women. Data were collected using the Introductory Information Form and the Theory-Based Intention for cesarean section scale. Analyses were performed using IBM SPSS 25.0 and AMOS 22.0. Language validity was evaluated through translation from the original Persian into Turkish as well as from English into Turkish. Content validity was calculated with nine experts using the Davis technique, and a pilot test was conducted with 30 pregnant women. Construct validity was assessed through Exploratory Factor Analysis and Confirmatory Factor Analysis. Internal consistency was examined with Cronbach's Alpha coefficient and test–retest reliability.

**RESULTS:**

Exploratory Factor Analysis revealed a five-factor structure with 24 items for the scale. According to Confirmatory Factor Analysis, the model fit indices indicated a good model fit. The Cronbach's Alpha coefficient of the scale was 0.76. The item–total correlations and test–retest reliability of the scale were found to be high.

**CONCLUSION:**

The Theory-Based Intention for cesarean section scale is a valid and reliable measurement tool for use among Turkish pregnant women.

## INTRODUCTION

Vaginal birth is a natural and physiological process, and medical interventions during this process are recommended to be kept to a minimum^1^. However, cesarean section (CS) may be necessary to ensure the well-being of the mother and the newborn. The World Health Organization (WHO) has reported that CS currently accounts for more than one-fifth (21%) of all births worldwide and is projected to rise to nearly one-third (29%) by 2030, highlighting the global increase in CS deliveries. Access to CS varies across regions of the world. While the CS rate is about 5% in Sub-Saharan Africa, in some countries, it has surpassed vaginal birth rates. In Turkey, CS rates have shown a rapid increase. With a rate of 50.8%, Turkey ranks among the top five countries worldwide with the highest CS rates^2^. Among 38 Organisation for Economic Co-operation and Development countries, Turkey holds the first place with a rate of 60%^3^.

CS plays a critical role in situations where vaginal birth may pose risks; therefore, access to CS should be ensured when necessary^2^. At this point, underuse of CS leads to increased maternal and perinatal mortality and morbidity, whereas overuse provides no additional benefits and results in the waste of workforce and financial resources^4–6^. The frequency of CS deliveries can be examined under three main categories: women's preferences, family- and society-related factors, and factors associated with healthcare professionals, healthcare systems, and institutional cultures^7^.

From the perspective of healthcare systems, low-quality services, lack of trust in antenatal care settings, equipment, or healthcare providers may increase the decision to perform CS. For healthcare professionals, fear of complications or malpractice during vaginal delivery, insufficient skills, and the relative ease of performing CS may contribute to its preference. From the women's perspective, negative previous experiences, fear of labor pain, concerns about pelvic floor damage, urinary incontinence, or potential negative effects on sexual health may incline them toward choosing CS. In addition, presenting CS as a controllable, convenient, or modern option may also influence women's decisions regarding delivery^7–10^.

Identifying changing birth preferences is important for reducing the increasing rates of CS over time. Predicting women's birth preferences in advance and planning appropriate interventions accordingly may help decrease CS rates^11^. At this point, there is a need for a valid and reliable measurement tool to reveal women's personal intentions regarding CS. This study aimed to examine the validity and reliability of the Theory-Based Intention for Cesarean Section (IR-TBICS) Scale, developed by Naghibi et al. in the Iranian population, in the Turkish language^12^. Validating the Turkish version will facilitate assessment of Turkish women's intentions regarding cesarean delivery.

## METHODS

### Type of study: This study employed a methodological design.

Study setting and timeframe: The research was conducted between September 22, 2023, and December 30, 2023, with pregnant women who applied to the obstetrics and gynecology outpatient clinic of a district hospital located in the Marmara Region of Turkey.

Population and sample: The study population consisted of pregnant women admitted to the outpatient clinic. Based on the recommendation of reaching at least ten times the number of scale items (24 items), a total of 300 pregnant women were included in the study^13^. Women between 28 and 40 weeks of gestation without high-risk pregnancies were included. Those with conditions preventing normal vaginal delivery or experiencing high-risk pregnancies were excluded.

Data collection tools: An Introductory Information Form and the TBICS Scale were used in the study.

Introductory ınformation form: This form consisted of 15 questions evaluating the demographic and obstetric characteristics of the participants^12,14^.

Theory-based ıntention for cesarean section scale: Developed by Naghibi et al. to determine the level of preference for cesarean delivery among pregnant women, the scale consists of 24 items and five subdimensions, rated on a five-point Likert scale. Higher scores indicate a stronger tendency to prefer cesarean delivery. The overall Cronbach's Alpha coefficient of the original scale was reported as 0.88^12^.

Data collection method: Data were collected using face-to-face interviews with a simple random sampling method. A pilot study was conducted with 30 pregnant women to evaluate the comprehensibility of the scale items, and the items were found to be understandable^15^. Pregnant women applying to the outpatient clinic were informed about the purpose and scope of the study, and those who agreed to participate signed an informed consent form. Participants completed the Introductory Information Form and the Intention for cesarean section scale, which took approximately 10 min. Two weeks later, the scale was re-administered to 30 participants to assess test–retest reliability.

Ethical considerations: Permission to conduct the Turkish validity and reliability study was obtained via e-mail from the corresponding author of the original version of the scale. The study was conducted in accordance with the Declaration of Helsinki. Ethical approval was obtained from the Kırklareli University Faculty of Medicine Scientific Research Ethics Committee (Decision No: 05, dated 12 December 2022). Institutional permission was also obtained. Informed consent was received from all pregnant women before data collection.

Data analysis: Data analysis was performed using IBM SPSS (Statistical Package for the Social Sciences) 25.0 and AMOS 22.0.

Validity analyses: Language validity was assessed by translating the scale from the original Persian into Turkish as well as from English into Turkish. The translations were performed by experts in Turkish language and literature (n=1) and English language and literature (n=1). Content validity was assessed by nine experts (four obstetricians, four academic midwives, and one clinical midwife) using the Davis technique. Content validity ratio and content validity index (CVI) were calculated. Item comprehensibility was further tested through a pilot study with 30 pregnant women. The adequacy and suitability of the sample for factor analysis were examined with Bartlett's test of sphericity and the Kaiser-Meyer-Olkin (KMO) test. Construct validity was assessed through Exploratory Factor Analysis (EFA) using the principal component method with Varimax rotation and Confirmatory Factor Analysis (CFA) by evaluating model fit indices.

Reliability analyses: Reliability was evaluated using Cronbach's Alpha coefficients and Pearson correlation coefficients for test–retest reliability.

## RESULTS

The ages of the pregnant women who participated in the study ranged from 19 to 40 years. In terms of education level, the highest proportion consisted of middle school graduates, accounting for 32.3% (n=97). Of the participants, 51% (n=153) were employed, and 70.7% (n=212) reported that their income was less than their expenses (Table 1).

**Table 1:** Sociodemographic characteristics of the participants (n=300).

		Minimum–maximum	Mean±SD
Age		19–40	28.59±5.11
**Variables**		**n**	**%**
Education level			
	Illiterate	42	14.0
	Primary school	88	29.3
	Secondary school	97	32.3
	High school	55	18.3
	University	18	6.0
Spouse's education level			
	Illiterate	8	2.7
	Primary school	44	14.7
	Secondary school	116	38.7
	High school	95	31.7
	University	37	12.3
Employment status			
	Employed	153	51.0
	Unemployed	147	49
Economic status			
	Income less than expenses	212	70.7
	Income equal to expenses	79	26.3
	Income more than expenses	9	3.0
Family structure			
	Nuclear family	263	87.7
	Extended family	37	12.3

SD: standard deviation.

The adequacy of the sample size and its suitability for factor analyses were assessed using the KMO coefficient and Bartlett's Test of Sphericity. The KMO value was calculated as 0.75, indicating that the sample size was adequate. Bartlett's test demonstrated that the dataset was suitable for factor analysis and was found to be significant [χ²(276)=4,665.25, p<0.001].

To evaluate the construct validity of the scale, an EFA was performed using the principal component method with Varimax rotation. The analysis revealed that 67.23% of the total variance was explained, and a five-factor structure with eigenvalues greater than 1, consistent with the original scale, was identified. Factor loadings ranged between 0.57 and 0.88, and all items were placed under the corresponding factors in line with the original version.

The Cronbach's Alpha coefficient for the total scale was calculated as 0.76, while the coefficients for the subdimensions ranged between 0.75 and 0.90. These results indicate that the scale demonstrates an adequate level of validity (Table 2).

**Table 2:** Factor structure and reliability results of the intention for cesarean section scale.

Items	F1 (outcome evaluations)	F2 (behavioral beliefs)	F3 (ınjunctive normative beliefs)	F4 (behavioral intention)	F5 (motivation to comply)	Cronbach's alpha
Item 8	0.85					0.89
Item 7	0.84					
Item 9	0.82					
Item 10	0.76					
Item 11	0.76					
Item 12	0.75					
Item 13	0.72					
Item 3		0.83				0.90
Item 4		0.82				
Item 1		0.82				
Item 2		0.81				
Item 5		0.80				
Item 6		0.66				
Item 18			0.74			0.75
Item 16			0.73			
Item 15			0.71			
Item 17			0.62			
Item 14			0.57			
Item 22				0.88		0.80
Item 24				0.85		
Item 23				0.81		
Item 20					0.86	0.88
Item 19					0.80	
Item 21					0.79	
Eigenvalue	4.61	4.13	2.70	2.45	2.22	
Explained variance (%)	19.24	17.24	11.26	10.22	9.25	

**Figure 1:**
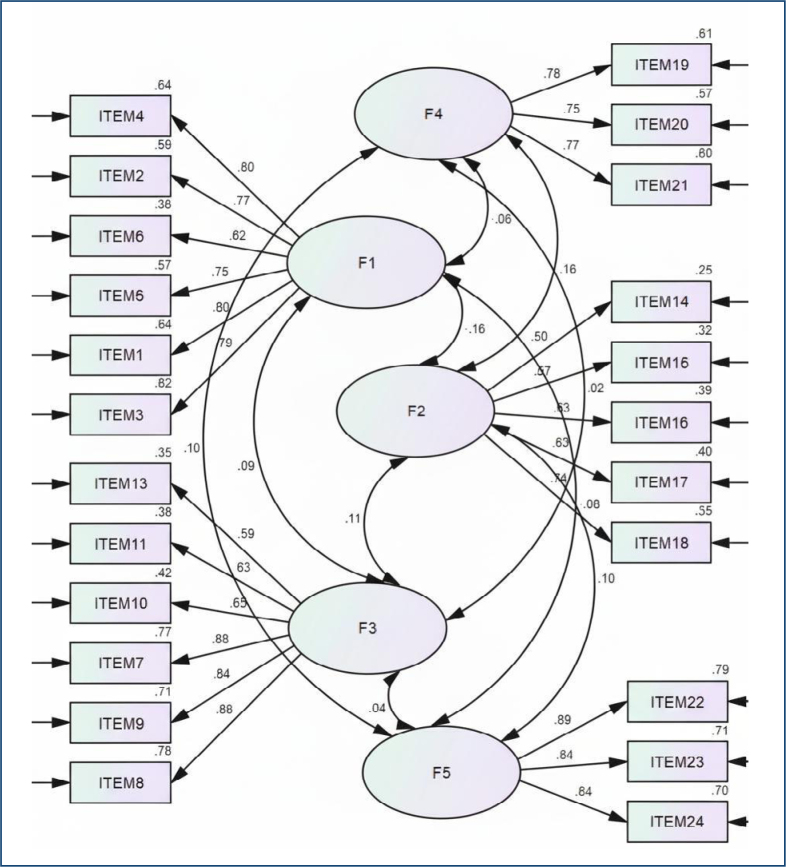
Results of the confirmatory factor analysis.

The results of the CFA showed that factor loadings for each item ranged from 0.50 to 0.89, all of which were statistically significant (p<0.001). Furthermore, the data demonstrated a good fit to the model, confirming the structure revealed by the EFA through model fit indices (χ²[220, n=300]=775.262, p>0.05, χ²/df=3.52, Goodness of Fit Index (GFI)=0.96, Adjusted Goodness of Fit Index (AGFI)=0.93, Tucker–Lewis Index (TLI)=0.96, Normed Fit Index (NFI)=0.95, Comparative Fit Index (CFI)=0.95, Root Mean Square Error of Approximation (RMSEA)=0.07) (Figure 1).

Test–retest reliability was used to assess the stability of the scale. Pearson correlation coefficients for all subdimensions and the total score ranged from 0.95 to 0.99 (p<0.001), indicating excellent temporal stability and confirming the scale's reliability.

Composite reliability (CR) values ranged from 0.79 to 0.93, and average variance extracted (AVE) values ranged from 0.53 to 0.68. Higher CR values indicate greater internal consistency, and AVE represents the amount of variance captured by each latent factor relative to measurement error (Table 3).

**Table 3:** Composite reliability and average variance extracted results of the intention for cesarean section scale.

Factor	CR	AVE
F1	0.92	0.68
F2	0.93	0.66
F3	0.79	0. 54
F4	0.84	0.53
F5	0.91	0.66

CR: composite reliability; AVE: average variance extracted.

Factor correlations ranged from 0.03 to 0.15 in absolute value. No correlation exceeded the 0.85 threshold typically associated with discriminant validity concerns (Table 4).

**Table 4:** Factor correlation matrix of the intention for cesarean section scale.

Factors	F1	F2	F3	F4	F5
F1	1.00				
F2	0.03	1.00			
F3	0.09	-0.15	1.00		
F4	0.09	-0.07	0.09	1.00	
F5	0.04	-0.03	0.12	0.08	1.00

## DISCUSSION

The rising rates of CS worldwide have become a significant public health concern, as they are associated with increased maternal and neonatal morbidity and healthcare costs^5,7^. In Turkey, CS rates are among the highest globally, emphasizing the need for reliable tools to understand and potentially influence women's birth preferences^2,3^. Identifying women's intentions regarding delivery methods is critical for planning interventions aimed at reducing unnecessary CS and promoting safe vaginal births.

The IR-TBICS Scale, originally developed by Naghibi et al., has undergone several Turkish adaptations. Previous studies by Calpbinici et al. and Taşkın et al. modified the number of items and subdimensions, likely due to regional differences in the samples^12,16,17^. In the validity and reliability study conducted by Calpbinici et al., data were collected from a province in the Central Anatolia Region. In this adaptation, the number of items was reduced to 20, while the number and names of the subdimensions remained unchanged^16^. In the study by Taşkın et al., data were collected from a province in the Eastern Anatolia Region, and the scale was structured with 18 items across three subdimensions. The original Intention for cesarean section scale consists of 24 items and five subdimensions^17^. In the present study, data were obtained from a district in the Marmara Region, and both the number of items and the subdimension structure were fully preserved as in the original scale. These variations across studies highlight the scale's flexibility while emphasizing that each adaptation provides a unique contribution to understanding Turkish women's intentions regarding cesarean delivery.

Initially, the content validity of the scale was evaluated by experts through translation from the original Persian version to Turkish, as well as from English to Turkish. Subsequently, the items were reviewed by nine field experts, and the CVI was calculated using the Davis method, resulting in a value of 0.96. In comparison, Naghibi et al. assessed content validity of the original scale using Lawshe's method with ten experts, including only items with a CVI of 0.62 or higher^12^.

Prior to data analysis, the adequacy of the sample size and the suitability of the data for factor analysis were examined using the KMO measure and Bartlett's Test of Sphericity. Both tests indicated that the sample size was sufficient and the dataset was suitable for analysis^18^.

Following content validation, construct validity of the Turkish-adapted scale was examined. EFA revealed that no item had a communalities value below 0.30 and the scale maintained a structure of 24 items and five factors consistent with the original version^13^. The variance explained by the subdimensions ranged from 9.25 to 19.24%, accounting for 67.23% of the total variance. For comparison, the original scale explained 62.46% of the variance^12^. In multi-factor scales, a total explained variance between 40 and 60% is generally considered acceptable (Table 2)^19^. These findings indicate that the Turkish version exhibits a structure similar to the original scale. CFA showed that factor loadings for items representing each factor ranged from 0.50 to 0.89, all statistically significant (Figure 1)^13^. Factor loadings above 0.30 indicate the suitability of the items for the structure^15^. In the CFA analysis, the RMSEA value was found to be 0.07. The literature states that an RMSEA value between 0.06 and 0.08 is an acceptable indicator of fit^20^. These findings indicate that the Turkish adapted scale performs well, confirming an excellent fit with the original structure.

Internal consistency was assessed using Cronbach's Alpha, which was calculated as 0.76 for the total scale. The subdimensions’ Cronbach's Alpha coefficients ranged from 0.75 to 0.90, compared to 0.60–0.84 in the original version^12^. Test–retest reliability was also evaluated, with item-total correlations ranging from r=0.95 (p>0.05), demonstrating stability over time^13^. Overall, the Turkish-adapted scale shows high internal consistency and temporal stability, comparable to the original scale.

The composite reliability and AVE results support the psychometric strength of the Turkish adaptation. CR values exceeded 0.70, indicating solid internal consistency while AVE values above 0.50 demonstrated adequate convergent validity. These indicators confirm that the constructs are measured reliably and that the items represent their underlying factors consistently^19,21^. The low factor correlations observed in the analysis indicate that the latent constructs are sufficiently distinct. None of the correlations approached the 0.85 criterion, providing evidence of discriminant validity and supporting the independence of the scale's subdimensions^19,21^.

### Limitations of the study

The findings are representative of women in the Marmara region; different birth preferences in other regions may influence the structure of the scale. The fact that the pregnant women included in the study were selected from a specific region and therefore had a specific sociocultural background weakens the study's ability to represent all of Turkish culture.

## CONCLUSION AND RECOMMENDATIONS

This study successfully adapted the IR-TBICS Scale, originally developed by Naghibi et al. in Iran, to the Turkish language and culture. The findings demonstrated that the Turkish version of the scale (TR-TBICS) has strong psychometric properties, including satisfactory construct validity and high internal consistency. Exploratory and Confirmatory Factor Analyses confirmed the five-factor structure, which aligns closely with the original scale, and the scale demonstrated excellent test–retest reliability, indicating temporal stability.

It is recommended to study the Turkish version of the scale across different sociocultural groups in Turkey and with a larger sample.

## Data Availability

The datasets generated and/or analyzed during the current study are available from the corresponding author upon reasonable request.
